# Ethnopharmacological evaluation of radal (leaves of *Lomatia hirsuta*) and isolation of 2-methoxyjuglone

**DOI:** 10.1186/1472-6882-6-29

**Published:** 2006-08-31

**Authors:** Henrik T Simonsen, Anne Adsersen, Louise Berthelsen, Søren B Christensen, Alfonso Guzmán, Per Mølgaard

**Affiliations:** 1 Department of Plant Biology, Royal Veterinary and Agricultural University, Thorvaldsensvej 40, DK-1871 Frederiksberg C, Denmark; 2 Department of Medicinal Chemistry, Danish University of Pharmaceutical Sciences, Universitetsparken 2, DK-2100 Copenhagen, Denmark

## Abstract

**Background:**

Leaves of *Lomatia hirsuta *are used in traditional medicine in Chile under the common name of "radal". A tea of radal is traditionally used for treatment of cough, bronchial troubles, and asthma. In a preliminary screening, extracts of the leaves revealed antifungal activity, and the present phytochemical study was undertaken to explain this activity and support the traditional use.

**Methods:**

Along with the traditional tea, extracts of the leaves were screened for antifungal and toxic activities. The profile of secondary constituents was obtained using GC-MS.

**Results:**

2-Methoxyjuglone was isolated from the leaves of *Lomatia hirsuta *and found to be active against the pathogenic fungus *Candida albicans *(MIC = 8 μg/mL). Cinnamic acid and vanillic acid were identified as major constituents in the tea by GC-MS. The tea was found not to be toxic against *Artemia salina*.

**Conclusion:**

The presence of phenolic acids with antimicrobial properties supports the traditional use of Radal, and encourages further studies.

## Background

*Lomatia hirsuta *(Lam.) Diels ex Macbr. (Proteaceae) is a wild tree growing in Chile from Coquimbo to Chiloe (IV-X Regions). It is distributed from the sea to sub mountain zones through 700–1200 m of altitude. Other *Lomatia *species in Chile are *L. ferruginea *(Cav.) R.Br. and L. *dentata *(R. et P.) R. Br. *L. hirsuta *is also present in Argentina, Ecuador and Peru [1]. Products prepared from the leaves of *L. hirsuta *are used in traditional medicine in Chile the product is under the common name of "radal". A tea of radal is used for treatment of cough, bronchial troubles, and asthma [2]. In a preliminary screening in our laboratory, methanol extracts of the leaves, but not of the stem, revealed antifungal activity, in particular against *Candida albicans *[3], and since our previous phytochemical study of the leaves did not explain the antifungal activity, the present study was undertaken. *C. albicans *and *Aspergillus fumigatus *were chosen as test organisms, since these organisms increasingly cause severe infections in patients with reduced immune response, e.g. HIV [4]. *Penicillium expansum *were also chosen as a test organisms, since this was a well establish culture in our lab.

An earlier study of *L. hirsuta *confirmed the presence of the hydroxycoumarins umbelliferone and scopoletin and the flavonoids quercetin, rhamnetin, iso-rhamnetin, and quercetrine [5]. An infusion of the leaves of *L. hirsuta *was found to possess a mild anti-inflammatory effect [5].

Naphthoquinones, such as lomatiol, juglone and naphthazasin, are the major metabolites in some *Lomatia *species [6], including native plants such as *L. ferruginea*, and *L. dentata*, but have not previously been reported from *L. hirsuta*.

## Methods

### Plant material

The leaves of *Lomatia hirsuta *(Lam.) Diels ex J.F. Macbr. (Proteaceae) were collected next to the river Gol-Gol east of Osorno, Chile. The plants were identified in the field by Alfonso Guzman and their identity verified by Professor Jaime Zapata from Universidad de Los Lagos, Osorno, Chile. Voucher specimens have been placed at Universidad de Los Lagos and at the Danish University of Pharmaceutical Sciences.

### Extraction and isolation

For the initial screening, 1 g of the dried leaf powder was ultrasonicated with 15 ml of methanol for 30 minutes. The extract was concentrated after filtration to give an average yield of 20% w/w. The active constituents were isolated from 320 g of dried leaves, which were extracted with first heptane and then methanol to give after vacuum evaporation of the solvent 1.5 % w/w and 9.3 % w/w of a residue, respectively. The residue of the methanol extract was partitioned between EtOAc and H_2_O and concentrated to yield residues of 7.3 g and 21.5 g, respectively.

The EtOAc phase (7.3 g) was subjected to VLC (300 g silica gel, 10 × 10 cm column) using CH_2_Cl_2 _(500 ml), CH_2_Cl_2_-EtOAc 19:1 to 1:1 (2500 and 1000 mL), neat EtOAc (1000 mL), and neat MeOH (1000 mL) as eluents. Repeated LC (using CH_2_Cl_2_-EtOAc, EtOAc, MeOH as eluents) of the CH_2_Cl_2 _fraction yielded 8.4 mg of 2-methoxyjuglone (**1**). The obtained ^1^H and ^13^C NMR spectra (Varian 300 MHz at 25°C, using TMS as standard) were similar to reported spectra of 2-methoxyjuglone [7].

The tea of the leaves was prepared by mixing 200 ml of boiling water with 20 g of dried leaves and leaving the mixture for 30 minutes. The mixture was filtered and the filtrate concentrated by lyophilisation to give a yield of 9.4 w/w %. For GC-MS study the tea was partitioned in an EtOAc fraction and a cold H_2_O fraction. Only the EtOAc fraction was analyzed in the GC-MS.

### Identification by GC-MS

Compound **1**–**8 **were identified in the biological active EtOAc fraction of the MeOH extract by GC-MS. The samples were dissolved in an appropriate solvent to c. 1 mg/mL. The GC (Agilent 6890N) inlet temperature was 200°C. The oven temperature was held at 50°C for 2 minutes and then with a rate of 20°C per minute increased to 300°C, which was held for 5 minutes. Total run time was 20 minutes with a constant flow of 1.2 ml/min. The column was a capillary column (Agilent 19091S-433), length 30 m, diameter 250 μm, film thickness 0.25 μm. The flow was split by 1:100 before introduction into the MS detector (Agilent 5973, electron impact (EI)): The EM voltage was 952.9 V, with lower and upper Mass limits set to 30 and 1000 *m/z*. Quadrupole temperature was 150°C and source temperature was 230°C.

The spectra were obtained by Enhancen ChemStation, MSD ChemStation D.01.02.16 provided by Agilent, compared with the spectra in the NIST/EPA/NIH Mass Spectral Library, Version 2.0 a ed. The library search was performed with the NIST Mass Spectral Search program against. All samples were analysed three times, and cinnamic and vanillic acids were run as standards to confirm the presence of these and similar compounds.

### Antimicrobial screening

A direct bioautographic method was used to determine the activity against *Penicillium expansum *(IMI 285521) and *Aspergillus fumigatus *(IBT 25732) [8]. A thin-layer chromatographic agar overlay technique [9] was used to determine the activity against *Candida albicans *(IMI 349010). These methods were used in the initial screening and the bio-guided fractionation. 100 μg of each fraction and extract were tested and the activity was observed visually. Amphotericin B was used as positive control for *C. albicans *(MIA = 1.2 μg) and nystatin for *P. expansum *(MIA = 0.5 μg).

A microplate method as previously described [10] was used with slight modifications to determine minimum inhibitory concentration values (MIC) of pure compounds against *C. albicans*. The compounds were dissolved in DMSO and diluted with Sabouraud broth (SAB) to a final DMSO concentration of 2 %. The plates were incubated for 48 h at 30°C and the growth of the fungi assessed after addition of MTT.

### Artemia salina toxicity assay

The *Artemia salina *(brine shrimp) toxicity assay was performed as previously described [11]. Six concentrations were tested in 96-well microplates in six fold, each well containing 10–20 nauplii.

## Results and discussion

A bioactivity guided fractionation lead to isolation of 8.5 mg of 2-methoxyjuglone (**1**) from a methanol extract of *Lomatia hirsuta*. In the antimicrobial assay with several organisms, the isolated 2-methoxyjuglone (**1**) was found to be active against *Candida albicans *with a MIC of 8 μg/mL, this is to the best of our knowledge the first report of antifungal activity of 2-methoxyjuglone. **1 **was also detected in our GC-MS analysis of the EtOAc fraction of the methanol extract. Derivatives of juglone are commonly found in genus *Lomatia*, but this is the first report of juglone derivatives in *L. hirsuta*. Species belonging to the Proteaceae are generally known to contain phenolic and caffeic acid derivatives and cinnamic acid (**2**) can be used as a chemical marker for Proteaceae [12]. Thus the identification of **2–8 **is not surprising when studying this family, but this is the first report of these compounds from this species.

Quinones (and among these naphthoquinones) are known for their antimicrobial but also toxic activity. In a recent report it was found that the activity could be referred to the orthoquinones (1,2 naphthoquinones) or paraquinones (1,4 naphthoquinones). The ortho was primarily antibacterial and the para mainly antifungal [13]. This is in good compliance with our observations where no antibacterial but antifungal activity was observed for 2-methoxyjuglone, which is a paraquinone.

The GC-MS analysis of the EtOAc fraction of the methanol extract confirmed the presence of cinnamic acid (**2**), ethyl cinnamate (**3**), 4-hydroxybenzoic acid (**4**), vanillic acid (**5**), methyl vanillate (**6**), isovanillic acid (**7**) and 4-hydroxyacetophenone (piceol) (**8**). Cinnamic acid was the major constituent in the crude methanol ethanol extract and the EtOAc fraction of this extract. None of the compounds **1**–**8 **have hitherto been identified in *L. hirsuta*. The presence of previously identified scopoletin and umbelliferone as major constituents [5] was confirmed by the GC-MS analysis. These two constituents were found in various fractions of the EtOAc fraction of the methanol extract. Since no antimicrobial activity was observed in the heptane extract and the H_2_O fraction of the MeOH extract, these extracts were not analyzed on GC-MS.

The initial screening revealed antifungal activity of the methanol extract against *C. albicans *and against *P. expansum*, whereas no antibacterial activity was seen, this activity was found again in the EtOAc fraction of methanol extract in our bio-guided isolation of **1**. The identification of **1 **– **8 **and the compounds reported in Erazo et al. [5], umbelliferone and scopoletin together with the flavonoids quercetin, rhamnetin, iso-rhamnetin, and quercetrine may explain the antifungal activity observed in the EtOAc fraction. In general phenolic acids (e.g. cinnamic acid), coumarins and flavonoids are known to have antimicrobial activities, all with different mechanism of actions and thereby different target organisms [14]. A literature study shows that **2 **and **3 **among several antimicrobial reports are reported to have broad antifungal activities [15] and that umbelliferone [16], scopoletin [17], **4**, **5 **and **7 **[18-21] have all been shown to have antifungal and/or antibacterial properties. The occurrence of this range of compounds could be part of the explanation of the antimicrobial effects seen in the initial study [3] and in the later fractionation work. Erazo et al. [5] showed that their extract had mild anti-inflammatory effect. Piceol (**8**) is known to possess anti-inflammatory properties in mice [22].

The tea of the leaves from *L. hirsuta *is locally used for the treatment of cough, bronchial troubles and asthma. **2 **and **4 **were identified in the EtOAc fraction of the tea by GC-MS, whereas none of the minor constituents was identified. **1 **and **3 **have previously been reported to be toxic [23,24]. But since high levels of juglones are previously reported only from the stem [13], the absence of **1 **and **3 **in the tea, makes it likely that the tea is non-toxic to humans. No toxicity of the tea against *Artemia salina *was demonstrated in a concentration range from 0.5 to 5 mg/ml, but it still remains to be established whether the tea could be toxic to human cells.

## Conclusion

This is the first report of the presence of the compounds **1–8 **in *Lomatia hirsuta*. Identification of 2-methoxyjuglone and the other compounds combined with the pharmacological test results encourage further studies on the effects of the tea of leaves of *L. hirsuta*. It also supports the traditional use of the plant.

## Competing interests

To the best of our knowledge there is no competing interest involved with the publication.

## Authors' contributions

HTS and LB performed the chemical laboratory work and the metabolite profiling. AA was involved in the screening of extracts for antifungal and toxic properties. SBC performed the structure elucidation of **1**. PM and AG performed the plant and data collection in Chile. All authors have read and approved the final manuscript.

**Figure 1:**
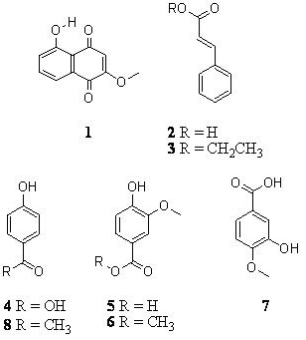
Identified compounds from *Lomatia hirsuta*. 2-methoxyjuglone (**1**) cinnamic acid (**2**), ethyl cinnamate (**3**), 4-hydroxybenzoic acid (**4**), vanillic acid (**5**), methyl vanillate (**6**), isovanillic acid (**7**) and 4-hydroxyacetophenone (piceol) (**8**).

## Pre-publication history

The pre-publication history for this paper can be accessed here:


